# Association of malaria and curable sexually transmitted infections with pregnancy outcomes in rural Burkina Faso

**DOI:** 10.1186/s12884-021-04205-6

**Published:** 2021-10-27

**Authors:** Serge Henri Zango, Moussa Lingani, Innocent Valea, Ouindpanga Sekou Samadoulougou, Biebo Bihoun, Diagniagou Lankoande, Phillipe Donnen, Michele Dramaix, Halidou Tinto, Annie Robert

**Affiliations:** 1 Pôle d’Epidémiologie et biostatistique, Université catholique de Louvain (UCLouvain), Institut de Recherche Expérimentale et Clinique (IREC), Clos Chapelle-aux-Champs, 30 bte B1.30.13, 1200 Brussels, Belgique; 2 Institut de Recherche en Sciences de la Santé, Direction Régionale du Centre Ouest (IRSS/DRCO), Nanoro, Burkina Faso; 3 Centre MURAZ, Institut National de Santé Publique (INSP), Bobo-Dioulasso, Burkina Faso; 4 École de santé publique, Université Libre de Bruxelles, CP594, route de Lennik 808, 1070 Bruxelles, Belgique

**Keywords:** Malaria, STI, Coinfection, Impact, Pregnancy, Outcome

## Abstract

**Background:**

Malaria and curable sexually transmitted infections (STIs) are severe infections associated with poor pregnancy outcomes in sub-Saharan countries. These infections are responsible for low birth weight, preterm birth, and miscarriage. In Burkina Faso, many interventions recommended by the World Health Organization were implemented to control the impact of these infections. After decades of intervention, we assessed the impact of these infections on pregnancy outcomes in rural setting of Burkina Faso.

**Methods:**

Antenatal care and delivery data of pregnant women attending health facilities in 2016 and 2017 were collected in two rural districts namely Nanoro and Yako, in Burkina Faso. Regression models with likelihood ratio test were used to assess the association between infections and pregnancy outcomes.

**Results:**

During the two years, 31639 pregnant women received antenatal care. Malaria without STI, STI without malaria, and their coinfections were reported for 7359 (23.3%), 881 (2.8 %), and 388 (1.2%) women, respectively. Low birth weight, miscarriage, and stillbirth were observed in 2754 (10.5 %), 547 (2.0 %), and 373 (1.3 %) women, respectively. Our data did not show an association between low birth weight and malaria [Adjusted OR: 0.91 (0.78 – 1.07)], STIs [Adjusted OR: 0.74 (0.51 – 1.07)] and coinfection [Adjusted OR: 1.15 (0.75 – 1.78)]. Low birth weight was strongly associated with primigravidae [Adjusted OR: 3.53 (3.12 – 4.00)]. Both miscarriage and stillbirth were associated with malaria [Adjusted OR: 1.31 (1.07 – 1.59)], curable STI [Adjusted OR: 1.65 (1.06 – 2.59)], and coinfection [Adjusted OR: 2.00 (1.13 – 3.52)].

**Conclusion:**

Poor pregnancy outcomes remained frequent in rural Burkina Faso. Malaria, curable STIs, and their coinfections were associated with both miscarriage and stillbirth in rural Burkina. More effort should be done to reduce the proportion of pregnancies lost associated with these curable infections by targeting interventions in primigravidae women.

## Background

Malaria and sexually transmitted infections (STI) are the most severe infections in pregnant women [1–3]. In sub-Saharan Africa, a study reported in 2017 that malaria was responsible for 217,019 stillbirth cases (20 % of all stillbirths) [4]. There was also an estimated 205,901 syphilis-related adverse pregnancy outcomes in 2015 [5]. In Burkina Faso, low birth weight (LBW) was at 13.4 % at the national level in 2010 [6]. In the same year, findings from a clinical trial showed a higher prevalence of LBW (18.1 %) in rural women who had malarial infections during their pregnancy [7]. The impact of STIs on pregnancy outcomes is less documented. A study conducted in Bobo-Dioulasso in Burkina Faso did not find an association between HIV infection and adverse pregnancy outcomes [8]. The impact of curable STIs such as syphilis, gonorrhea, chlamydia, or trichomoniasis on pregnancy outcomes has not been studied in Burkina Faso yet. Data on these infections are however routinely collected in health facilities in Burkina Faso [9]. This lack of information could result in a lack of funding for the prevention of these curable STIs. Malaria associated with STIs during the same pregnancy could cause more adverse pregnancy outcomes than a single infection. Our study aimed to assess the association between malaria and curable STIs routinely diagnosed -and pregnancy outcomes in rural settings of Burkina Faso.

## Methods

### Settings

Burkina Faso is classified as a low-income country in sub-Saharan Africa. In 2020, the total population was estimated at 20 million inhabitants, predominantly rural population (69.4 %). The birth rate is estimated at 35.1 births/1000 population, with a maternal mean age of 19.4 years at first birth. Malaria is endemo-epidemic and is the first cause of consultation, accounting for 41.3 % of all consultations in 2018 in health facilities [10, 11].

### Study design

We retrospectively collected data from all antenatal consultations and deliveries during two consecutive years in 61 health facilities of the districts of Nanoro and Yako in rural Burkina Faso. Pregnant women who delivered between January 1, 2016, and December 31, 2017 were considered in our study. Details of the full methodology is described elsewhere [9]. In brief, individual patient information are manually recorded in registers by health care providers (nurses and midwives) in health facilities during antenatal care visits and at delivery. According to the national guidelines, pregnant women are requested to attend monthly antenatal care visits in the health facility they belong to, for clinical management of their pregnancy. These visits occur from the first trimester to delivery and six weeks thereafter. They receive monthly oral sulfadoxine-pyrimethamine for malaria prevention in accordance with intermittent preventive treatment (IPT-SP). They are encouraged to deliver at the health facility under the assistance of the nurse or midwives. For complicated cases, women are referred to the hospitals’ districts.

### Definitions

In all settings of Burkina Faso, pregnancy outcomes were defined as recommended by the World Health Organization (WHO). Miscarriage was a pregnancy lost before 28.0 weeks of gestation. Stillbirth was defined as fetal death at 28 weeks or more of gestation or with a birth weight of at least 1000 g [12, 13]. Preterm birth was defined as a baby born alive from 28.0 weeks to less than 37.0 weeks of gestation. LBW was defined as a birth weight less than 2500 g in a singleton live newborn with at least 37.0 weeks of gestation. Congenital infection meant that the baby was born with an infection whatever the etiology. Poor outcome means that the woman experienced at least one of the following adverse outcomes: preterm, miscarriage, stillbirth, or have delivered a baby with low birth weight or a congenital infection.

Malaria is diagnosed using rapid diagnostic tests or microscopy when available, in symptomatic pregnant women during the antenatal care visit. Positive cases are treated with artemisinin-based combination namely artemether-lumefantrine, artesunate-amodiaquine, or quinine. Curable STIs are managed using clinical syndrome-based algorithms.

Syphilis screening is also routinely performed during the first antenatal visit using Treponema Pallidum Hemagglutinations Assay in the medical centers at the peripheral level of the health system. The body mass index was calculated using the height and the weight measured at the delivery or during the last antenatal visit performed.

### Statistical analysis

Data are reported as means with standard deviation when continuous and as numbers with proportion when categorical. Associations of malaria and curable STIs with LBW, miscarriage, and stillbirth were assessed using logistic regression analysis with likelihood-ratio tests. Only the factors associated with pregnancy outcome with p-value < 0.05 in the univariate analysis were considered in the multivariate regression. IPT-SP was not included in the adjustment of the relationship between miscarriage and infections because IPT-SP was not administered before 16 weeks of gestation when the early miscarriage occurred. The odds ratio and adjusted odds ratio were reported with their 95 % confidence interval. All analyses were performed using Stata® version 16.0 StataCorp LLC, Texas, USA.

### Ethics

The study protocol was approved by both the National Ethics Committee in Burkina Faso (Comité d’Ethique pour la recherche en Santé, CERS) with the registration number: 2018-6-076 and the “Comité d’Ethique Hospitalo-Facultaire Saint-Luc UCLouvain” in Belgium with the registration number: 2018/14MAR/109. All data were anonymized.

## Results

### Characteristics of study participants

**Fig. 1:**
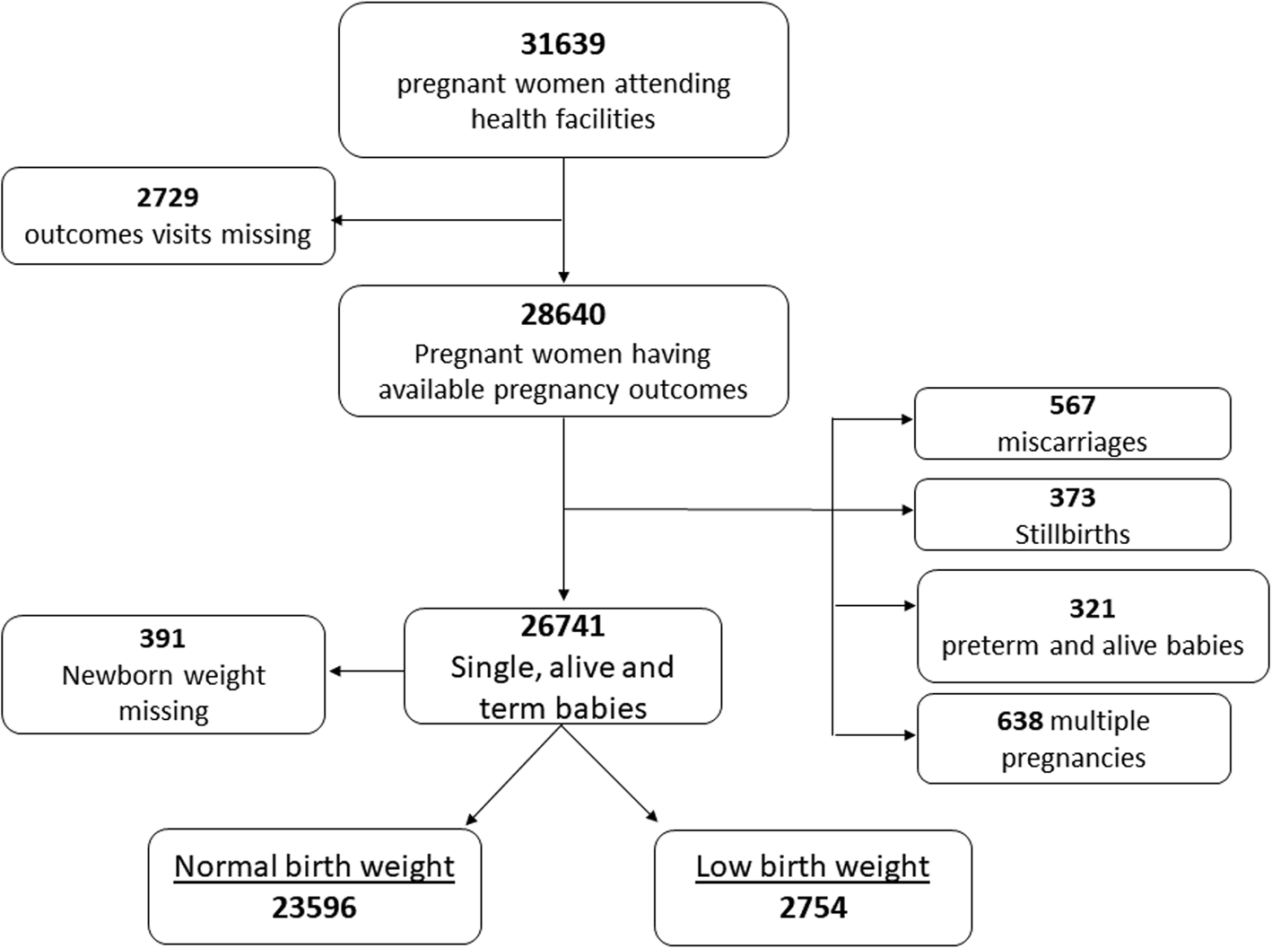
Flow chart of pregnant women and delivery outcomes from 2016 to 2017

Data from 31639 pregnant women who attended health facilities between 1^st^ January 2016 and 31^st^ December 2017 were considered. Out of these, 2729 (8.6 %) had no data on the delivery visit and 26741 newborns were single and alive at term (Fig. 1). Table 1 shows the characteristics of the study participants. The proportion of teenagers (under 20 years) was 20.9 %. The mean body mass index at delivery was 22.9 ± 2.7 kg/m^2^ with 2085 (6.6 %) ≤ 20 kg/m^2^. Women who received no IPT-SP represented 14.2 %.

**Table 1:** Characteristics at delivery in a cohort of 31369 consecutive pregnant women in Nanoro and Yako

	Mean ± SD	n (%)
**Age (Yrs)**	25.4 ± 6.3	
< 20		5568 (20.9)
20 - 34		18120 (67.9)
≥35		2984 (11.2)
Missing		4967
**Gravidity**		
Primigravidae		6288 (22.7)
Secundigravidae		9297 (33.6)
Multigravidae		12093 (43.7)
Missing		3961
**Mother's BMI (kg/m**^**2**^**)**	22.9 ± 2.7	
≤ 20		2085 (6.6)
> 20		29554 (93.4)
Missing		0
**IPT-SP**		
None		2965 (14.2)
1-2 doses		9755 (46.8)
≥ 3 doses		8141 (39.0)
Missing		10778
**Infections**		
Malaria and STI		388 (1.2)
Malaria without STI		7359 (23.3)
STI without malaria		881 (2.8)
Neither malaria nor STI		23011 (72.7)
Missing		0

*BMI* Body mass index, *IPT-SP* intermittent preventive treatment with sulfadoxine-pyrimethamine

Out of the 31639 pregnant women, 338 (1.2 %; 95 % CI: 1.1 – 1.4 %) had a coinfection of malaria and STIs, 7359 (23.3 %; 95%CI: 22.8 – 23.7 %) had symptomatic malaria without curable STI, and 881 (2.8 %; 95%CI: 2.6 – 3.0 %) had curable STIs detected by symptoms without malaria.

### Outcomes prevalence

The prevalence of LBW, miscarriage, and stillbirth in pregnant women were 2754 (10.5 %; 95%CI: 10.1 – 10.8 %), 547 (2.0; 95%CI: 1.8 – 2.1 %) and 373 (1.3 %; 95%CI: 1.2 – 1.4 %), respectively. Preterm birth and congenital infection prevalence were 321 (1.1 %; 95% CI: 1.0 – 1.2 %) and 180 (0.6 %; 95%CI: 0.5 – 0.7 %), respectively (Table 2).

**Table 2:** Delivery outcomes in 31369 pregnant women in Nanoro and Yako from 2016 to 2017

	Mean ± SD	n (%)
**Birth weight (g)**	2910 ± 457	
**Low birth weight (< 2500g)**		
Yes		2754 (10.5)
No		23596 (89.5)
Missing		5289
**Preterm and alive**		
Yes		321 (1.1)
No		28351 (98.9)
Missing		2967
**Congenital infection**		
Yes		180 (0.6)
No		28492 (99.4)
Missing		2967
**Miscarriage**		
Yes		567 (2.0)
No		28105 (98.0)
Missing		2967
**Stillbirth**		
Yes		373 (1.3)
No		28299 (98.7)
Missing		2967
**Poor outcome**^a^		
Yes		4408 (15.6)
No		23791 (84.4)
Missing		3440

^a^women having at least one out of the five poor outcomes in preceding lines

### Factors associated with poor outcomes

Table 3 reports univariate and multivariate associations of women characteristics and LBW. LBW was higher but not significantly associated with coinfections of malaria and STIs (12.9 %): adjusted OR= 1.15 (0.75 - 1.78), and symptomatic malaria without STIs (10.6 %): adjusted OR= 0.91 (95% CI: 0.78 - 1.07) compared to women without malaria nor STIs. LBW was lower but not significantly associated with curable STIs detected by symptoms without malaria (9.8 %): adjusted OR= 0.74 (95% CI: 0.51 - 1.07), compared to women without malaria nor STIs.

**Table 3:** Relationship between women infection and low birth weight without and with adjustment

		Low birth weight				
	n	%	OR (95%CI)	*P*-Value	Adj OR (95% CI)	*P*-Value
					*N*=17467	
**Maternal infections**				0.55		0.22
Malaria and STI	280	12.9	1.23 (0.89 - 1.81)		1.15 (0.75 - 1.78)	
Malaria without STI	4420	10.6	1.03 (0.92 - 1.14)		0.91 (0.78 - 1.07)	
STI without malaria	542	9.8	0.93 (0.70 - 1.24)		0.74 (0.51 - 1.07)	
Neither malaria nor STI	21108	10.4	1		1	
**Age (yrs)**				< 0.001		0.65
< 20	4445	13.9	1.53 (1.39 - 1.70)		1.06 (0.94 - 1.20)	
20 - 34	14915	9.5	1		1	
≥35	2495	8.9	0.92 (0.80 - 1.07)		1.02 (0.87 - 1.21)	
**Gravidity**				< 0.001		< 0.001
Primigravidae	5772	21.1	3.74 (3.40 - 4.13)		3.53 (3.12 - 4.00)	
Secundigravidae	8654	8.1	1.23 (1.11 - 1.37)		1.19 (1.04 - 1.35)	
Multigravidae	11159	6.7	1		1	
**Mother’s BMI (kg/m**^**2**^**)**				< 0.001		< 0.001
≤ 20.0	1975	17.0	1.86 (1.64 - 2.11)		1.86 (1.60 - 2.17)	
> 20.0	24677	9.9	1		1	
**IPT-SP**				< 0.001		< 0.001
None	2042	12.9	1.41 (1.22 - 1.64)		1.40 (1.19 - 1.64)	
1-2 doses	8839	10.2	1.08 (0.98 - 1.20)		1.08 (0.97 - 1.20)	
≥ 3 doses	7433	9.5	1		1	

*BMI* Body mass index, *IPT-SP* intermittent preventive treatment with sulfadoxine-pyrimethamine, *NI* not included, *Adj OR* adjusted odd ratio

Table 4 presents univariate and multivariate associations of women characteristics and preterm birth. Preterm birth was higher but not significantly associated with coinfections (1.3 %) and symptomatic malaria (1.3 %), adjusted OR: 1.32 (95% CI: 0.42 - 4.19) and 1.42 (95% CI: 0.97 - 2.07), respectively. Preterm birth was lower but not significantly associated in women experiencing STIs detected by symptoms without malaria (0.7 %), adjusted OR: 0.70 (95% CI: 0.22 - 2.21) compared to women without malaria nor STIs.

**Table 4:** Relationship between women infection and low preterm birth without and with adjustment

		Preterm birth				
	n	%	OR (95%CI)	*P*-Value	Adj OR (95% CI)	*P*-Value
					*N *= 18731	
**Maternal infections**				0.48		0.28
Malaria and STI	305	1.3	1.20 (0.44 - 3.24)		1.32 (0.42 - 4.19)	
Malaria without STI	4856	1.3	1.17 (0.88 - 1.54)		1.42 (0.97 - 2.07)	
STI without malaria	592	0.7	0.61 (0.23 - 1.66)		0.70 (0.22 - 2.21)	
Neither malaria nor STI	22919	1.1	1		1	
**Age (yrs)**				0.002		0.69
< 20	4836	1.5	1.67 (1.26 - 2.21)		1.13 (0.81 - 1.58)	
20 - 34	16233	0.9	1		1	
≥35	2758	1.1	1.22 (0.83 - 1.80)		0.92 (0.55 - 1.53)	
**Gravidity**				< 0.001		<0.001
Primigravidae	6264	2.2	2.99 (2.28 - 3.91)		3.14 (2.21 - 4.45)	
Paucigravidae	9258	0.9	1.22 (0.90 - 1.64)		1.07 (0.72 - 1.59)	
Multigravidae	12058	0.7	1		1	
**Mother’s BMI (kg/m**^**2**^**)**				0.23		NI
≤ 20.0	2076	1.1	1.28 (0.87 - 1.87)		NI	
> 20.0	26596	1.4	1			
**IPT-SP**				0.03		0.14
None	2417	1.5	1.74 (1.16 - 2.61)		1.48 (0.95 - 2.30)	
1-2 doses	9460	1.2	1.30 (0.96 - 1.77)		1.30 (0.95 - 1.78)	
≥ 3 doses	7892	0.9	1		1	

*BMI* Body mass index, *IPT-SP* intermittent preventive treatment with sulfadoxine-pyrimethamine, *NI* not included, *Adj OR* adjusted odd ratio

**Fig. 2:**
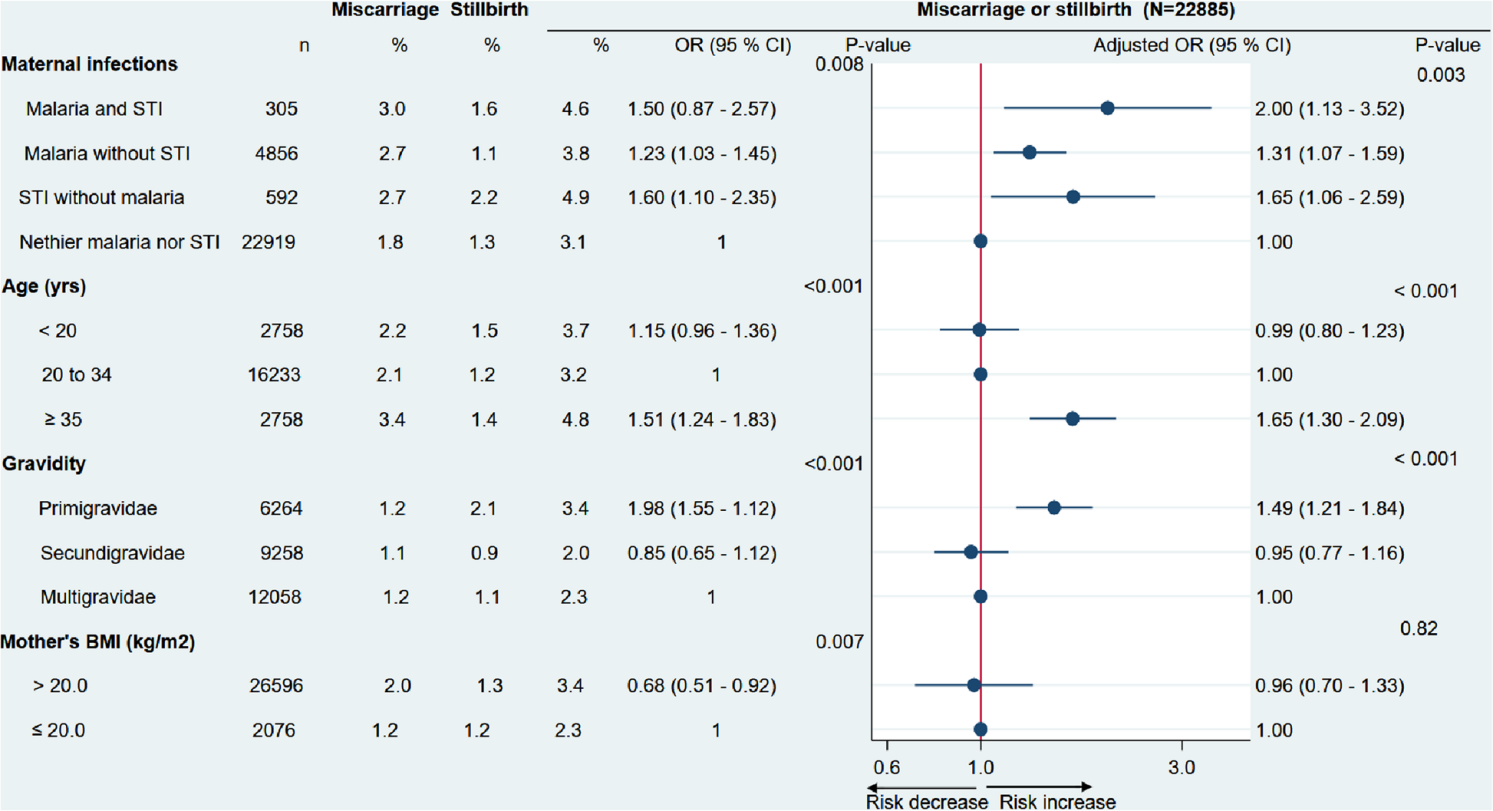
Univariate and multivariate odds ratio of both miscarriage and stillbirth in pregnant women from Nanoro and Yako health districts. The red solid vertical line is the reference line. Black dots are the estimated odds ratio. Horizontal black lines are 95 % confidence intervals. An estimate is not significant when its confidence interval crosses the red line. BMI: body mass index. STI: sexually transmitted infections

Figure 2 shows a forest plot of maternal factors associated with miscarriage or stillbirth. Miscarriage and stillbirths were higher in women with a coinfection of malaria and STIs than in women without infection (4,6 vs 3.1 %); adjusted OR: 2.00 (95% CI: 1.13 - 3.52)

Miscarriage and stillbirths were also higher in women experiencing malaria without STI (3.8 %) and in women with STI without malaria (4.9 %) compared to women without infections; adjusted OR: 1.31 (95% CI: 1.07 - 1.59) and 1.65 (95% CI: 1.06 - 2.59), respectively.

## Discussion

Our study aimed to assess the impact of symptomatic routinely diagnosed malaria and STIs on pregnancy outcomes, in the rural settings of Burkina Faso.

Our results show that LBW was still frequent in rural settings of Burkina Faso. This prevalence was slightly lower compared to the prevalence at national level in 2010 and supports a sustained trend of decrease of LBW in Burkina Faso, as suggested by He et al*.* in 2018 [14]. Bihoun et al*.* in 2017 reported a higher prevalence of LBW (18.1%) in pregnant women with malaria infection between 2010 and 2012 in the same area [7]. The subgroup of women with malaria in our study had a lower prevalence of LBW (10.6 %). The increase of IPT-SP doses from 2 doses to three doses at least, according to the revised national guidelines in 2014 probably had an impact. Also, we considered malaria infections in women with clinical symptoms irrespective of the gestational age, contrary to the study of Bihoun et al*.* that considered only pregnant women in the second and third trimester attending the health facilities. Asymptomatic malaria (not diagnosed in our study) could cause LBW as previously reported by Cottrell et al*.* in 2015 [15] and could also explain the difference in the prevalence. LBW was not associated either with malaria nor with STIs in our study. This probably reflects the effect of the various efforts being deployed to control these infections. The effective management of malaria cases with the introduction of rapid diagnostic tests to improve the diagnosis of malaria and treatment with efficacious artemisinin-based combinations since 2014 in Burkina Faso have probably contributed to reducing the burden of malaria in pregnancy. Previous studies suggested that malaria infections occurring in the third trimester of gestation contribute more to the occurrence of LBW [16]. So, the IPT-SP administered monthly from the sixteenth week of gestation for the prevention of malaria contributes to reducing the occurrence of LBW. Furthermore, SP has an antibiotic property on STIs, which probably contributes to reducing the effect of these infections on birth weight [17–20]. LBW is still frequent in the rural settings of Burkina Faso but it is not associated with symptomatic STI nor malaria in pregnant women.

The prevalence of LBW decreased with the gravidity in our study based on multivariable analysis, suggesting that interventions to prevent LBW could have a better impact if they focus on primigravidae mothers. Primigravidae are known to be associated with LBW by the mechanism of placental malaria as described in many studies [21–24]. Our study was not able to report placental malaria, and this can be considered a limitation in assessing this association.

The proportion of LBW was lower in mothers with BMI < 20 kg/m^2^ in our study. Several studies reported the same association in other settings [7, 25–27]. In Burkina Faso, maternal low BMI in the rural setting is often caused by nutritional deficiency [28]. Both macronutrient’ and micronutrient deficiency leads to a disruption of maternal and fetal exchanges and alters the modulating of oxidative stress, inflammation, and enzyme function. These mechanisms disturb fetal growth which results in LBW [29, 30]. In our study, 6.6 % of women had a low BMI measured at delivery, and this rate could be underestimated because of gestational weight gain [26, 31]. Nutritional supplementation interventions were implemented in the rural health facilities in Burkina Faso, but their impact seems to remain suboptimal. Their uptake by the beneficiaries can be questioned. Comprehensive monitoring and evaluation of nutritional programs being implemented could provide key information’s on their strengths and weaknesses in view of identifying improvement opportunities.

The prevalence of preterm births (1.1 %) in our study was slightly lower than what was reported by Scott et al*.* in 2019 in west Africa, including Burkina Faso (3.0 %) [32]. However, this prevalence was not significantly associated with malarial nor STI infections. As the measure of symphysis-fundal height for gestational age can be user-dependent, a difference in the proportion can be observed when rigorous and standardized procedures are not in place. Only primigravidae were strongly associated with preterm births after adjustment. Additional analyses which consider asymptomatic infections could provide betters estimates on the association between infections and preterm births.

Symptomatic malaria, curable STIs, and their coinfections were significantly associated with both miscarriages and stillbirths in this study. This was not the case in Zambia as reported by Chaponda et al*.* in 2016 [1]. In their study, only infections diagnosed in the first antenatal care visit were considered, thus ignoring the effect of infections occurring later during the pregnancy. Our analysis considered only symptomatic infections. From the available evidence, it is not clear if symptomatic infections could have more impact on pregnancy outcomes than asymptomatic ones. The pregnancies lost were more associated with coinfections than malaria only and STI only in our study. These infections probably have a synergetic action on pregnancy outcomes suggesting that in addition to preventing malaria, the prevention of STIs is of importance in the limited resource settings. Strategies combining IPT-SP to antibiotics such as azithromycin have shown potential benefits to prevent poor pregnancy outcomes because this antibiotic has also antimalarial properties against Plasmodium falciparum [19, 20]. However, more evidence-based studies are still needed. Therapeutic prevention of infections in the first trimester could reduce the number of pregnancy loss but data on adverse reactions of most drugs still need to be collected to inform on their safety [33]. Family planning intervention could also be beneficial toward primigravidae and women above 35 years who were also more associated with pregnancies lost in our study.

### Limitations and strengths

The use of routine data to assess infections’ impact on the pregnancy outcomes in a constrained resource setting could induce some bias. In most cases, malaria diagnosis was performed by using rapid diagnostic tests that have low sensitivity and specificity [34]. The diagnosis of STIs performed by using clinical algorithms has a low sensitivity and a low specificity [35, 36]. However, this is also data used by the Ministry of health for decision-making.

At antenatal care, the gestational age was mainly obtained by using symphysis-fundal height that has low precision. It was however the only possibility we had to differentiate miscarriage from stillbirths. By mixing both miscarriages and stillbirths as pregnancy lost in the analyses, we lifted the limitation related to the precision of symphysis-fundal height measurement. We were not able to get data on placental malaria as this is not routinely available. We had less than 10 % missing delivery visits, but missing information could have impacted the analysis given the small number of some pregnancy outcomes.

## Conclusion

Pregnancy losses contrary to low birth weights and preterm birth were associated with symptomatic malaria, curable STIs detected by symptoms, and their coinfections in the rural setting of Burkina Faso. Sulfadoxine-pyrimethamine used in prevention in the last decade contributed to reducing LBW. More effort should be done to reduce the proportion of pregnancies lost associated with these curable infections by targeting interventions in primigravidae women.

## Data Availability

The dataset used for analysis in this study is available from the corresponding author and will be provided on a reasonable request.

## Abbreviations

Adj OR: Adjusted odds ratio
BMI: Body mass index
CERS: Comité d’Ethique pour la Recherche en Santé
CI: Confidence interval
HIV: Human immunodeficiency virus
IPT-SP: Intermittent preventive treatment with sulfadoxine-pyrimethamine
LBW: Low birth weight
SD: Standard deviation
STI: Sexually transmitted infections
UCLouvain: Université catholique de Louvain
WHO: World Health Organisation
